# Isolation, Identification, and Characterization of Phosphate-Solubilizing Bacteria from Tunisian Soils

**DOI:** 10.3390/microorganisms11030783

**Published:** 2023-03-18

**Authors:** Marwa Amri, Mohamed Ridha Rjeibi, Marwa Gatrouni, Dina M. R. Mateus, Nedra Asses, Henrique J. O. Pinho, Chaabane Abbes

**Affiliations:** 1 Laboratory of Resources Sylvo-Pastoral, Institute Sylvo-Pastoral of Tabarka (ISPT), Université de Jendouba, Isp.Tabarka BP. n° 345, Tabarka 8110, Tunisia; 2 Laboratory Research of Science and Technology of Environmental (LRSTE), Higher Institute Science and Technology Environmental À Borj Cédria, and Faculty of Sciences of Bizerte (FSB), Université de Carthage, BP-1003, Hammam-Lif 2050, Tunisia; 3 Laboratoire de Parasitologie, École Nationale de Médecine Vétérinaire de Sidi Thabet, Université de La Manouba, Sidi Thabet 2020, Tunisia; 4 Laboratoire de Parasitologie, Institut de La Recherche Vétérinaire de Tunisie, Université de Tunis El Manar, 20 Rue de Jebel Lakdhar, La Rabta, Tunis 1006, Tunisia; 5 Techn&Art, Centre for Technology, Restoration and Art Enhancement, Instituto Politécnico de Tomar, Estrada da Serra, 2300-313 Tomar, Portugal; 6 Laboratory of Ecologies and Microbial Technology (LETMI), National Institute of Applied Science and Technology (INSAT), Université de Carthage, 2 Boulevard de La Terre, B.P. 676, Tunis 1080, Tunisia; 7 Ci2, Smart Cities Research Center, Instituto Politécnico de Tomar, Estrada da Serra, 2300-313 Tomar, Portugal

**Keywords:** indole acetic acid, phosphate-solubilizing bacteria, phosphate solubilization index, tricalcium phosphate

## Abstract

Soil microorganisms play an important role in maintaining natural ecological balance through active participation in carbon, nitrogen, sulfur, and phosphorous cycles. Phosphate-solubilizing bacteria (PSB) are of high importance in the rhizosphere, enhancing the solubilization of inorganic phosphorus complexes into soluble forms available for plant nutrition. The investigation of this species of bacteria is of major interest in agriculture, as they can be used as biofertilizers for crops. In the present study, 28 isolates of PSB were obtained after the phosphate enrichment of soil samples from five Tunisian regions. Five PSB species were identified by 16S rRNA gene sequencing including *Pseudomonas fluorescens*, *P. putida*, and *P. taiwanensis*, *Stenotrophomonas maltophilia*, and *Pantoea agglomerans*. Solid and liquid Pikovskaya’s (PVK) and National Botanical Research Institute’s (NBRIP) media containing insoluble tricalcium phosphate were used for the evaluation of the phosphate solubilization ability of the bacterial isolates by two methods: visual evaluation of the solubilization zone around colonies (halo) and determination of solubilized phosphates in liquid medium by the colorimetric method of the vanado-molybdate yellow. Based on the results of the halo method, the isolate of each species that showed the higher phosphate solubilization index was selected for evaluation of phosphate solubilization by the colorimetric method. In the liquid media, the bacterial isolates showed phosphate solubilization ranging from 535.70 to 618.57 µg mL^−1^ in the NBRIP medium, and 374.20 to 544.28 µg mL^−1^ in the PVK medium, with the highest values produced by *P. fluorescens*. The best phosphate solubilization ability and higher reduction in broth pH, which indicates higher organic acid production, were achieved in NBRIP broth for most of the PSB. Strong correlations were observed between the average capability of PSB to solubilize phosphates and both the pH and total phosphorous content in the soil. The production of the hormone indole acetic acid (IAA), which can promote plant growth, was observed for all five PSB species. Among them, *P. fluorescens* obtained from the forest soil of northern Tunisia showed the highest production of IAA (50.4 ± 0.9 µg mL^−1^).

## 1. Introduction

Phosphorus (P) is considered the most important element in plant nutrition, after nitrogen [1]. It represents an essential component in all main metabolic pathways of plants such as photosynthesis, signal transduction, and respiration [2]. However, the concentration of soluble P in soil is usually very low (400–1260 mg kg^−1^) [3], which requires the use of fertilizers to ensure higher crop productivity to respond to the increasing demand because of the continuous growth of the world population. For this reason, the overall need for P fertilizers has been increasing by 2.5–3.0% per year [4].

Conventional chemically obtained fertilizers include various forms of P, such as triple superphosphate, mono-ammonium phosphate, di-ammonium phosphate, and ammonium polyphosphate. The major problem with the application of chemical fertilizers is that a large part of the soluble form of inorganic phosphate applied to the soil is rapidly immobilized and becomes unavailable to plants [5]. Additionally, higher availability of P in the soil can reduce the assimilation of heavy metals, such as cadmium, mitigating the risk of negative effects on plant growth as well as animal and human health through the food chain [6].

The scarcity of phosphorous world reserves is another essential aspect that motivates the search for solutions to prevent the low availability of phosphorous in soils as a result of its immobilization. World phosphorous reserves are being depleted because of the use of P in several industries, but mainly for the production of fertilizers [7]. In addition to the depletion of phosphorus resources by the phosphate- and phosphoric acid-producing industries, which are relevant in Tunisia, the production processes generate large amounts of waste that are creating serious environmental problems. This issue is evidenced in the Gulf of Gabes, in southern Tunisia [8]. Therefore, the use of strategies to reduce fertilizer consumption, such as improving their effectiveness through higher solubilization of phosphates in soils, can contribute to reducing the negative impacts of phosphorus industries.

Solubilization of mineral phosphates is an important ability exhibited by some microorganisms, motivating the study of their solubilizing capability [9]. The phosphate-solubilizing bacteria (PSB) are predominant over other phosphorus-solubilizing microorganisms and play an important role in biogeochemical phosphorus cycling in both terrestrial and aquatic environments [10].

PSB can solve the problem of the ability to solubilize soil-insoluble phosphates through different mechanisms, such as the secretion of organic acids, the production of enzymes, and the excretion of siderophores [11]. In addition, PSB can promote plant growth through the production of hormones such as auxins, cytokinins, and gibberellic acid, as well as ethylene, hydrogen cyanide, and siderophores [12]. PSB’s contribution to nitrogen fixation and resistance to soil pathogens is also reported as promoting plant growth [13].

PSB are part of the plant growth-promoting rhizobacteria and can solubilize different inorganic phosphate compounds, such as tricalcium phosphate and other mineral phosphates, allowing for a reduction in the use of synthetic fertilizers in agriculture [14]. Therefore, the use of PSB as a bioinoculant is considered an ecological and sustainable approach [15].

Considering that the solubilization of phosphates in fertilizers and other phosphorous compounds already present in the soil is a contribution to the sustainable use of resources, the purpose of this work was to study various species of PSB found in different Tunisian soils and to assess the strains which are the most effective in the solubilization of phosphates. In addition, as some bacteria were reported to produce hormones and other compounds that promote plant growth, and the potential of the isolated bacteria for producing the hormone indole acetic acid was also evaluated. Studying the PSB in the Tunisian soils has two main justifications: Tunisia is a relevant example for the development of sustainable management of phosphorous compounds, thus mitigating the environmental impact of phosphate production; and using locally available PSB already adapted to Tunisian soil types is a way to expand their application in the country.

## 2. Materials and Methods

### 2.1. Soil Samples

#### 2.1.1. Site Description

A series of sampling was carried out in two types of soils (forest soil and agricultural soil) in the area of Tabarka near a hydrothermal source in Aïn Draham (Hammam Bourguiba). The site location and characteristics are shown in Table 1.

#### 2.1.2. Sampling Design

Three samples were collected at each location. The samples were taken at a depth of 15 cm with a hand auger and placed in sterile polythene bags with appropriate labeling, stored in a cooler, and brought to the laboratory for further analysis.

#### 2.1.3. Determination of Soil Physical and Chemical Parameters

In the laboratory, the samples were homogenized, sieved (through a 2 mm mesh screen), and stored at 4 ± 0.1 °C in a refrigerator. The samples were then subjected to various analyses, such as pH, organic matter (OM), total carbon (C_Total_), total nitrogen (N_Total_), total phosphorus (P_Total_), and texture determination.

The pH was measured after suspending 20 g of soil in 40 mL of distilled water. The OM was determined by sample weight loss on ignition at 600 °C, after removal of moisture by drying at 60 °C for 24 h. The C_Total_ was estimated by dividing the OM value by 1.724 [16]. The N_Total_ was determined using the Kjeldahl method following the mineralization of soil samples at 400 °C after the addition of sulfuric acid and cupric sulfate. The P_Total_ was determined by the vanado-molybdate yellow method after the digestion of soil samples with hydrochloric acid. The soil texture analysis was performed according to Robinson’s pipette method as described by Mwendwa [17].

### 2.2. Isolation and Purification of Phosphate Solubilizing Bacteria

As a first step to isolate the PSB, 10 g of sample soil was added to 140 mL of phosphate selective media (90 mL of sterile water and 50 mL of nutrient broth containing insoluble sources of P such as AlPO_4_, FePO_4_, and phytate), and then were agitated at 130 rpm in an orbital shaker at 30 °C for 7 days [18]. From this suspension, a dilution series ranging from 10^−1^ to 10^−5^ was carried out. A volume of 1 mL of each dilution was then spread on a plate count agar medium PCA (containing 5 g/L of tryptone, 2.5 g/L yeast extract, 1 g/L glucose, and 12 g/L agar) (surface seeding) and 1 mL was placed at the bottom of a Petri dish (deep seeding). After incubation at 30 °C for 24 h, PSB were isolated from soil samples by serial dilution using plating on PCA medium. This step was carried out by successive subculturing until a homogeneous culture was obtained and all the colonies became identical.

### 2.3. Identification of the Studied Isolates

The following morphological characteristics were used for the identification of isolated bacteria: shape, size, mobility, presence or absence of spores, and Gram staining.

The molecular identification of isolated bacteria was performed after DNA extraction carried out by thermal lysis. From a young culture of 24 h on agar medium, colonies were suspended in an Eppendorf tube with 200 μL of sterile distilled water, incubated at 100 °C for 10 min, and finally centrifuged at 14,000 rpm for 10 min. The supernatant containing the DNA was collected and stored at −20 °C until the PCR analysis.

A PCR was run using universal primers (forward EU49f [5′-TTAACACATGCAAGTCGAACGG-3′], reverse EU1070r [5′-GGACTTAACCCAACATCTCACGA-3′]) that amplify a 1021 bp sequence of 16S rRNA [1]. The reaction was performed in a final volume of 25 µL, containing 1× PCR buffer, 1.5 mM MgCl_2_, 0.3 mM dNTPs and 1.4 units of Taq DNA Polymerase (Biobasic Inc., Markham, ON, Canada), 10 pmol of each primer, and 1 µL of DNA template.

The thermocycling was performed with a thermocycler (ESCO Swift MaxPro, Horsham, PA, USA) and started with an initial denaturation for 5 min at 94 °C, followed by 30 cycles (94 °C for 60 s, 55 °C for 60 s and 74 °C for 60 s) and a final extension at 74 °C for 5 min. PCR products were separated by electrophoresis in 1.2% agarose gel to evaluate the size of the amplicons.

All positive amplicons obtained with the primers EU49f/EU1070r were purified with the Wizard SV gel and PCR clean-up system (Promega, Madison, WI, USA) according to the manufacturer’s instructions. Purified DNA fragments were sequenced in both directions using the same primers as for PCR. The reactions were performed using a conventional Big Dye Terminator cycle sequencing-ready reaction kit (Applied Biosystems, Foster City, CA, USA) with an ABI3730XL automated DNA sequence.

The obtained 16S rRNA gene sequences were edited using the ChromasPro software (version 1.7.4). The pairwise nucleotide percent identity of the new sequences was calculated using MEGA 6.1 software [19]. Phylogenetic trees were constructed by the neighbor-joining method. Distances were estimated by the Tamura-Nei method [20].

### 2.4. Solubilization of Phosphates

The bacteria isolates were tested for their ability to solubilize phosphate according to the methods of Nautiyal [21] and Pikovskaya [22] using the NBRIP and PVK media. An improved Pikovskaya’s phosphate growth (PVK) solid medium containing blue bromophenol, which produced yellow-colored halos around the colonies due to organic acid production, was developed by Gupta et al. [23], and is commonly used in the screening of PSB [9]. Nautiyal [21] developed the National Botanical Research Institute’s phosphate growth liquid medium (NBRIP) comprising glucose, 5.0 g/L of tricalcium phosphate (TCP), magnesium chloride hexahydrate, magnesium sulfate heptahydrate, potassium chloride ammonium sulfate, and distilled water, and this medium is widely used for the evaluation of phosphate solubilization [9,24].

The ability of bacteria to solubilize the TCP was determined by measuring the solubilization halo diameter around the colonies after the inoculation of fresh bacterial suspension on the PVK and NBRIP agar media. The solubilization index (SI) was calculated after 7, 14, and 21 days of incubation at 28 ± 2 °C using the following formula [25]:

$$\mathrm{SI}=\frac{\mathrm{CD}+\mathrm{HD}}{\mathrm{CD}}$$

where CD is the colony diameter, and HD is the halo zone diameter.

The phosphates’ solubilizing capacity of the PSB isolates was also evaluated based on the colorimetric measurement of the concentration of solubilized phosphates in liquid medium. Fifty mL of liquid medium (NBRIP or PVK) supplemented with 0.5% TCP was inoculated with 200 μL of fresh bacterial suspension with an optical density of 0.8, corresponding to 5 × 10^8^ CFU/mL, and incubated at 28 ± 2 °C for 7 days on a rotary shaker at 180 rpm [26]. Afterwards, the cultures were centrifuged at 10,000 rpm for 10 min, and the supernatant was used for the quantification of solubilized phosphorus by the colorimetric method of vanado-molybdate yellow at 430 nm [27]. The pH of the broth medium was also measured with a digital pH meter (Jenway 3510) at three and seven days of incubation.

The two methods described above were applied sequentially: first, all isolates from soil samples were screened for their SI; then, the isolate of each species that showed better effectiveness (SI ≥ 2) was tested by the colorimetric method. All experiments were carried out in triplicate for each isolate.

### 2.5. Determination of the Production of Indole Acetic Acid

PSB were assayed for their capacity to produce indole acetic acid (IAA) in Lauria Bertani (LB) medium containing L-tryptophan as a precursor (10 g/L), yeast extract (5 g/L), and NaCl (10 g/L). Fifty ml of LB medium was inoculated with one mL of bacterial suspension (10^9^ CFU/mL) and incubated at 28 ± 2 °C for 4 days in a shaking incubator at 180 rpm. Then, 4 mL of Salkowski reagent (prepared from 50 mL of 35% perchloric acid and 1 mL of 0.5 M FeCl_3_ solution) was added to 1 mL of the supernatant obtained by centrifuging the bacterial suspension at 6000 rpm for 30 min [28]. The ability to produce IAA was indicated by the appearance of pink color after the addition of Salkowski reagent. Absorbance was measured at 530 nm and the quantity of IAA was determined from a standard curve and expressed as μg/mL. These experiments were carried out in triplicate for each isolate.

### 2.6. Data Analysis

The experimental data were organized and analyzed using Microsoft Excel^®^ spreadsheet software, version 2206, 64 bit. This spreadsheet software was also used to calculate the means and the 95% confidence intervals of phosphate-solubilizing activity, pH, and IAA production.

IBM SPSS^®^ software package version 28 was used to perform Pearson’s correlations, Analysis of Variance (ANOVA), and post hoc comparison tests with a confidence level of 95% (α = 0.05). ANOVA F-test test was performed to determine the significance of the differences between the means of phosphate-solubilizing activity, pH, and IAA production, and Tukey’s HSD post hoc method was applied to identify homogeneous subsets. Pearson correlation analysis was performed to determine the correlation between PSB’s phosphate-solubilizing activity and the soil’s physicochemical properties.

## 3. Results and Discussion

### 3.1. Physical and Chemical Characteristics of Soil Samples

The physical and chemical characteristics of the soil samples are shown in Table 2. All sites had soil with a sandy texture and almost neutral pH. The soil from site 5 had the lowest organic matter and carbon content. The phosphorus was is similar for all soils and close to the value of 70 ppm.

### 3.2. Characterization of the Isolates

Twenty-eight bacterial colonies were isolated and their macroscopic and microscopic characteristics were defined. Isolated colonies had pink and yellowish colors. All bacteria had stem form and Gram-negative bacterial group identity, and they were mobile and spore-free. Meireles et al. [29] reported that this type of bacteria is most abundant in soils. According to Patten and Glick [30], these rod-shaped, motile, non-spore-forming bacteria with Gram-negative strains usually belong to the genus *Pseudomonas*, which is predominant in the rhizosphere.

### 3.3. Molecular Identification and Phylogenetic Analyses

**Figure 1:**
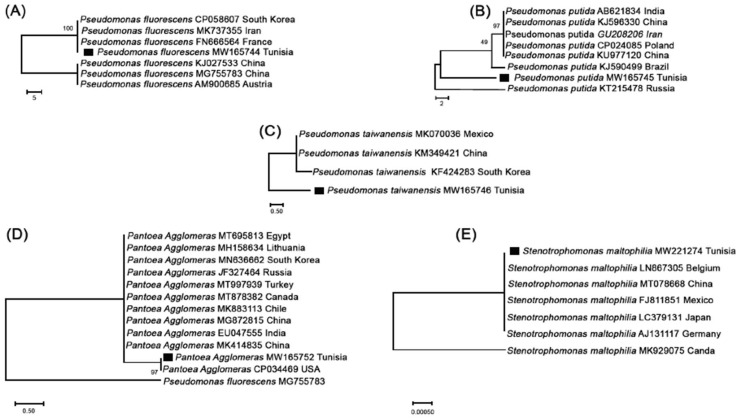
Partial sequence 16SrRNA gene phylogenetic tree of *Pseudomonas* (**A**–**C**), *Pantoea agglomerans* (**D**), and *Stenotrophomonas* (**E**) species. (Species described in this study are indicated with a black square).

The molecular identification of 28 isolates confirmed the presence of five species belonging to three genera, namely *Pseudomonas* (*P. fluorescens*, *P. putida*, and *P. taiwanensis*), *Pantoea agglomerans*, and *Stenotrophomonas maltophilia* (Figure 1). The finding of these five species of PSB is in line with the previous results stating that bacteria belonging to the genera *Pseudomonas*, *Enterobacter*, and *Bacillus* [31], *Pantoea* [32], in addition to some species of the genera *Rhizobium* and *Arthrobacter* [33,34], are powerful PSB. Biswas et al. [31] mentioned that *Pseudomonas* spp., *Bacillus* spp., and *Rhizobium* spp. are among the most effective genera to solubilize phosphates. In addition, strains of the genus *Pantoea* have been specified as solubilizing agents. Singh and Jha [35] also demonstrated that *Stenotrophomonas maltophilia* is a powerful species in the solubilization of P, which makes it possible to release phosphate from insoluble granules such as tricalcium phosphate or other natural phosphates.

### 3.4. Composition of PSB Community

**Figure 2:**
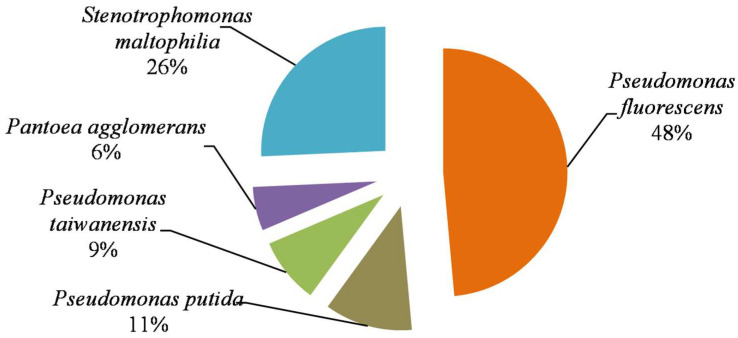
Relative abundance of the different species of PSB in the studied Tunisian soils.

Among isolated PSB, *P. fluorescens* dominated, followed by *S. maltophyla*, *P. putida*, *P. taiwanensis*, and *P. agglomerans* (Figure 2). Similar results were obtained by Meireles et al. [29], stating that the genus *Pseudomonas* is estimated as the dominant PSB in soils.

### 3.5. Calculation of the Phosphate Solubilization Index

All isolated colonies were screened for phosphate solubilization on solid media. The phosphate solubilization ability is marked by the formation of transparent halos around the bacterial colony (on the solid PVK and NBRIP media), supplemented with TCP as the only source of phosphorus. The isolates showed a clear halo zone around their colonies, which could be the result of the production of organic acids or polysaccharides, or the activity of phosphatase enzymes [10]. The average SI of the most effective isolate of each specie is presented in Table 3.

The difference in the SI means was statistically significant among the results obtained for both NBRIP and PVK media (based on the one-way ANOVA, *p* < 0.01). Moreover, SI of the same isolates on different media were also significantly different (*p* < 0.01). This observation confirms that the halo method should be limited to a preliminary screening of PSB, although being a practical step because of its its easy and fast application [10]. For this reason, the isolated and purified bacteria were also assayed in a liquid medium with a precise quantification of soluble phosphorus released to validate their activity.

Still, SI can be a basis for a preliminary comparison of the five isolates’ ability to solubilize TCP. Thus, the isolates of the genus *Pseudomonas* seem to be the most efficient for solubilizing solid phosphates among the tested isolates, although differences among them were not statistically significant based on the one-way ANOVA. The solubilization of solid phosphates is a very common characteristic in *Pseudomonas* and certain *Enterobacteriaceae* (including *P. agglomerans*) [32]. These bacteria also play a significant role in the soil through their metabolic activities, for example, the solubilization of complex forms of phosphate to more accessible forms for plants and through the processes of acidification, chelation, and exchange reactions [36].

The isolates of *Stenotrophomonas maltophilia* and *Pantoea agglomerans* showed contradictory results on SI in the two solid media, but the average SI for both species is significatively lower than the SI of the isolates of the *Pseudomonas* genus. However, isolates of *S. maltophila* and *P. agglomerans* can be considered effective PSB. Previously, Ramos et al. [37] showed that *S. maltophilia* can grow on various phosphate-selective media. The findings of the present work are also supported by the results of Kumar and Audipudi [28], which demonstrated that *S. maltophilia* AVP27 isolated from pepper rhizosphere was able to produce prominent areas of P solubilization on the PVK agar plate. On the other hand, *Pantoea* is a less-studied genus, but some of their species, such as *P. agglomerans*, have already been described as PSB [38].

### 3.6. Evaluation of the Phosphate Solubilization in Liquid Media

**Figure 3:**
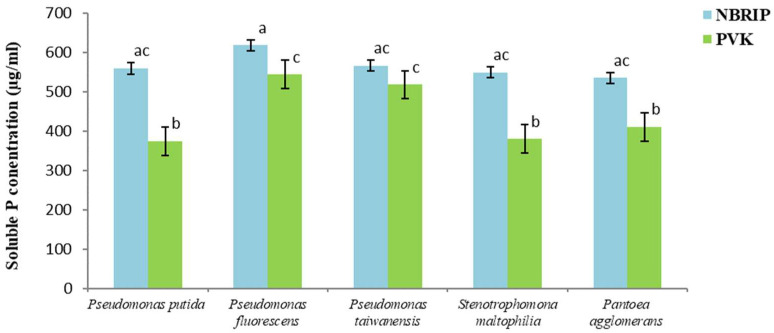
Phosphate solubilization by bacterial isolates grown in NBRIP and PVK media at 28 ± 2 °C. Vertical bars represent confidence intervals at a confidence level of 95% (n = 3). Same letters indicate that differences among the means is not statistically significant (Tukey’s HSD post hoc test).

Figure 3 shows the results obtained for the five isolates in liquid NBRIP and PVK media. Although the solubilization levels in liquid media varied among different isolates, all the isolates were capable of solubilizing TCP as the only source of phosphate in PVK and NBRIP media. The differences between the mean solubilization ability in the two culture media are statistically significant according to the one-way ANOVA (*p* < 0.01).

The phosphate-solubilizing ability of the tested isolates in NBRIP liquid medium, with TCP as a source of insoluble phosphorus, varied from 535.70 to 618.57 µg mL^−1^, with maximal ability demonstrated by *P. fluorescens*. In the liquid medium PVK, the solubilization of TCP by the PSB varied from 374.20 to 544.28 µg mL^−1^, and *P. fluorescens* also demonstrated the best solubilization capability.

The highest phosphate solubilization was observed in the medium NBRIP for all isolates. These results are in accordance with the results of Nautiyal et al. [39], who classified the liquid NBRIP medium as the best medium for testing the phosphate solubilization. Cherif et al. [40] and Chibani et al. [41] also reported that NBRIP is the preferred liquid medium for phosphate solubilization by the majority of rhizobacteria.

The isolates of *P. fluorescens* produced the highest concentration of soluble phosphate in the NBRIP broth after 7 days of incubation (618.57 µg mL^−1^). This finding is supported by the results obtained by Gupta et al. [42] and Ahmad et al. [43], showing that *Pseudomonas* sp. is an effective phosphate solubilizer. The isolates of *S. maltophilia* and *P. agglomerans* also exhibited a high level of phosphate solubilization capability in the liquid NBRIP. The values obtained are even higher than the values obtained by Patel and Saraf [44] and Son et al. [38], 362 µg mL^−1^ for *S. maltophilia* and 200 µg mL^−1^ for *P. agglomerans*.

The solubilization of phosphate by the isolates in the PVK liquid media showed significant differences in their means. Moreover, all PSB but *P. taiwanensis* showed statistically lower solubility in PVK medium than in NBRIP medium. In the PVK medium, *P. fluorescens* and *P. taiwanensis* had a significatively higher phosphate solubilization capability than the other three isolates. Although the results obtained in both liquid media are promising, further studies must be conducted in the soil to validate the effective capability of those PSB in real field conditions.

### 3.7. pH Changes in NBRIP and PVK Liquid Media

**Figure 4:**
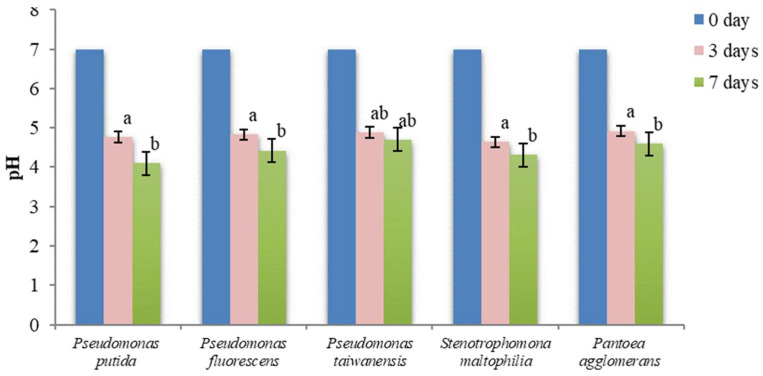
Variation of pH in NBRIP culture medium. Vertical bars represent confidence intervals at a confidence level of 95% (n = 3). Same letters indicate that differences among the means is not statistically significant (Tukey’s HSD post hoc test).

**Figure 5:**
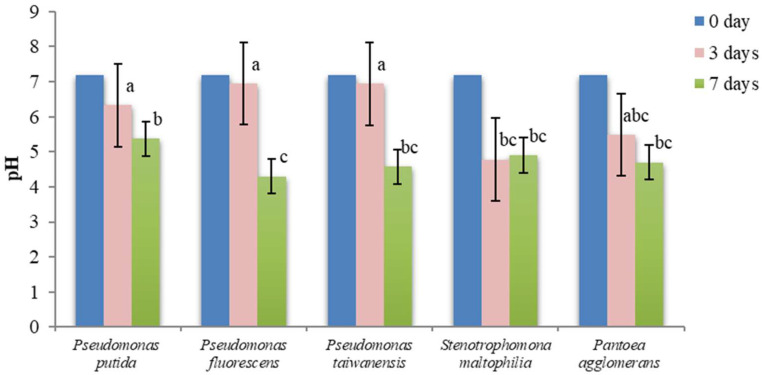
Variation of pH in PVK culture medium. Vertical bars represent confidence intervals at a confidence level of 95% (n = 3). Same letters indicate that differences among the means is not statistically significant (Tukey’s HSD post hoc test).

The solubilization of TCP by PSB was accompanied by a significant decrease in the pH of the culture supernatant, both in the NBRIP and PVK media (Figure 4 and Figure 5).

All values were significantly different from the initial pH (*p* < 0.01, one-way ANOVA) in NBRIP culture medium. The means were significatively different for the pH at three and seven days (*p* < 0.01), but were not significatively different among different species (*p* = 0.18 and 0.19, respectively for three- and seven-day values, from the one-way ANOVA).

Zhu et al. [45] proved that the solubilization of insoluble phosphate depends on various factors but, in particular, is accompanied by a decline in pH. In fact, the results of the present work confirmed a decrease in pH value during the TCP solubilization by all five strains of PSB, like in the NBRIP medium where final pH values ranged from 4.1 to 4.7.

The solubilization of phosphate in PVK medium was also accompanied by a decrease of the original pH (Figure 5), as observed for the NBRIP medium. However, the mean decreases of pH observed for the first three days of cultivation in the PVK medium were not statistically different from the initial pH for the three *Pseudomonas* species (*p* = 0.23), being significantly different after seven days (*p* < 0.01). The final pH after the seven days of culture ranged from 4.3 to 5.4, higher than the pH observed for the cultures in the NBRIP medium.

Several works reported a similar decrease in the culture media pH during phosphate solubilization: Park et al. [46] and Muleta et al. [47] reported that *P. flurescens* reduced the pH values of media to 4; Ghosh et al. [48] reported a decrease in the pH from 7 to 4.3 after four days of *S. maltophila* incubation in NBRIP medium; Costa et al. [49] reported a decrease to pH 5 after the cultivation of *P. agglomerans* in PVK medium.

The acidification of culture supernatants indicated that the production of organic acids seemed to be the main mechanism for phosphate solubilization. Wan et al. [50] reported that various PSB produced gluconic acid, 2-ketogluconic acid, lactic acid, and citric acid during the solubilization of insoluble phosphates. Khan et al. [51] and Oteino et al. [52] also showed that the solubilization activity of PSB in the soil is based on the secretion of organic acids. In addition, the production of acids to release phosphorus from mineral complexes by various genera of bacteria including *Bacillus*, *Pseudomonas*, *Enterobacter*, *Serratia*, and *Azotobacter*, in connection with other chemical mechanisms such as chelation reactions, was also demonstrated by Kishore et al. [36].

When comparing the medium acidification with the average phosphate concentration in liquid media, a strong negative correlation was observed in PVK medium (Pearson’s correlation coefficient of −0.854, *p* = 0.065). On the other hand, a very weak correlation was seen in the NBRIP media (Pearson’s correlation coefficient of −0.049, *p* = 0.938). This unexpected result is in parallel with the observation that the final pH values are not statistically different across all five PSB cultures in the NBRIP medium, giving the hypothesis that a pH near 4 can be a lower limit value for the growth of the isolated PSB in phosphate-rich media. However, future work needs to be carried out to confirm this hypothesis.

### 3.8. Determination of Indole Acetic Acid Production

**Figure 6:**
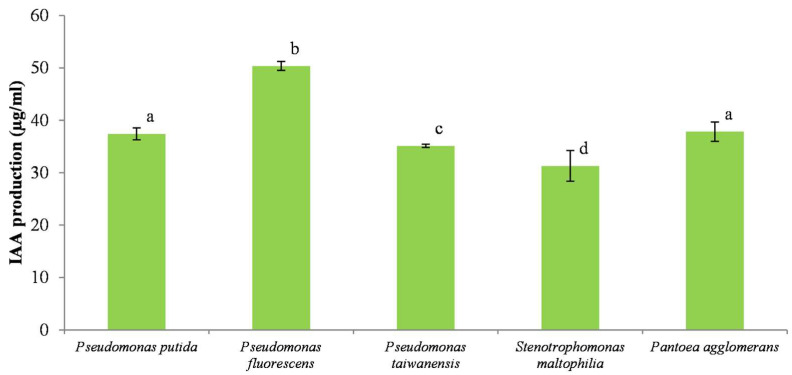
IAA production (µg/mL) by bacterial isolates. Vertical bars represent confidence intervals at a confidence level of 95% (n = 3). Same letters indicate that differences among the means is not statistically significant (Tukey’s HSD post hoc test).

Indole acetic acid production was characteristic of almost all bacterial isolates. The IAA production ranged from 31.29 to 50.37 µg mL^−1^, as shown in Figure 6. A relatively high content of IAA was found in the culture of *P. fluorescens* (50.37 µg mL^−1^), followed by *Pantoea agglomerans* and *P. putida* with 37.83 and 37.41 µg mL^−1^, respectively.

Endogenously produced or exogenously applied organic substances are known as regulators of plant growth and development [53,54]. They are active in a very low concentration and affect several morphological and physiological processes. Among the different types of plant growth regulators, IAA has important functions in cell proliferation, apical dominance, tropic responses, and reproduction [11]. Various rhizobacterial strains have been reported to produce IAA in significant amounts and help in plant growth promotion.

A high level of IAA production by *Pseudomonas* species was also revealed in the studies of Ahmad et al. [43] and Kumar et al. [55]. In addition, species from the genus *Stenotrophomonas* were also found to effectively produce IAA in the culture medium both in the presence and absence of tryptophan. Suckstorff and Berg [56] found that *S. maltophilia* produced 31.29 µg mL^−1^ of IAA. However, Singh and Jha [35] reported that for another strain of the same species (SBP-9) produced only 3.16 µg mL^−1^ of IAA.

Many PSB are reported as plant growth promoters, with their beneficial effects on plants including alleviation of nutrient deficiency via phosphorus solubilization [57]. Stimulation of disease resistance mechanisms (inducing systemic resistance) and ground pathogen protection are also reported as beneficial effects of PSB [58]. The production of siderophores which solubilize and sequester iron from the rhizospheric environment is another reported contribution of PSB [59]. Thus, localized nutrient supply (especially P) can greatly stimulate root and shoot proliferation.

### 3.9. Correlation with Soil Properties

The five PSB species identified in this work had a different abundance in each type of soil, and not all species were found in all five locations. *P. fluorescens*, *P. putida*, *P. taiwanensis*, *P. agglomerans*, and *S. maltophilia*, in this order, were predominant in the locations numbered one to five described in Section 2.1. Thus, it is possible to evaluate the correlation between soil properties and the phosphate’s solubilization capacities of the studied PSB. The correlation of the average solubilized phosphate concentration in liquid media with soil properties is shown in Table 4.

Strong correlations were found between phosphate solubilization by PSB and pH and total phosphorous concentration in the soil. However, only the correlation with the total phosphorous was statistically significant. The correlations with the remaining soil properties were weak. All correlations were positive except the correlation with soil pH.

PSB isolated from soils with lower pH showed higher phosphate solubilization potential. The correlation with the phosphorus concentration in the soil was very strong and positive. The higher the concentration of phosphorous in the soil, the greater the solubilization capacity of species isolated from that soil.

These results point to an apparent adaptation of the PSB species to soil pH and phosphorous concentration. These results are coherent with the observed trend of pH decrease with the increasing capacity of PSB species to solubilize phosphates.

## 4. Conclusions

PSB contribute to the high availability of soluble phosphates which can be assimilated by plants, mitigating the issue of the immobilization of a large fraction of the inorganic phosphates applied to the soil through chemical fertilizers. Soil inoculation with PSB can be a direction towards the better use of phosphates for plant fertilization, since phosphorous is a scarce element and the industrial production of phosphoric acids and phosphates causes severe environmental pollution. Therefore, the selection of PSB with high capabilities of phosphate solubilization is a green and sustainable way to make phosphates effectively available for plant growth.

In this work, 28 bacterial isolates belonging to five species from soils of five Tunisian locations were screened for phosphate solubilization. Three species of the genus *Pseudomonas* (*P. putida*, *P. fluorescens*, and *P. taiwanensis*) showed the highest capability in solubilizing phosphates. *P. fluorescens* was the most effective, attaining a soluble phosphate concentration of 618.57 µg mL^−1^ in NBRIP phosphate-enriched liquid culture medium. Although not as effective, *Stenotrophomonas maltophilia* and *Pantoea agglomerans* also showed appreciable phosphate-solubilizing capabilities.

Strong correlations were observed between the phosphate-solubilizing capabilities of the studied PSB and pH and total phosphorous content of the sampled soils, negative and positive, respectively. The decrease in pH is attributed to the secretion of organic acids by the PSB that contribute to phosphate solubilization.

The highest secretion of indole acetic acid was produced by *P. fluorescens* (50.37 µg mL^−1^), but all five isolates of PSB species showed significant values of IAA production. This hormone is reported as essential for plant growth; thus, its production by PSB is another argument for their use as a soil bioinoculant.

The application of locally available PSB can be a way to improve Tunisian soils while reducing the use of phosphate fertilizers, which can also therefore contribute to diminishing the prejudicial effects of the Tunisian phosphoric acid industry. However, the results obtained can be useful for any region in which agriculture depends on phosphate fertilization.

Further work can focus on understanding the observed differences of cultivating PSB in different media, but more valuable work should be carried out in real field conditions. Hence, the evaluation of the real and long-term effect of PSB on plant assimilation of nutrients and plant growth is needed to validate the large-scale and commercial valorization of using isolated PSB.

## Figures and Tables

**Table 1:** Geographical location and characteristics of the sampling sites.

Site	Location	Climate	Average Temperature	Typical Monthly Precipitation	Main Culture	Type
1	N: 36°46′12.0″ E: 008°34′44.5″	Warm temperate	18.6 °C	1–11 mm	Hackberry trees	Forest
2	N: 36°46′10.7″ E: 008°34′41.2″	Warm temperate	18.6 °C	1–11 mm	Quercus suber	Forest
3	N: 36°46′12.6″ E: 008°34′42.6″	Warm temperate	18.6 °C	1–11 mm	Hackberry trees	Forest
4	N: 36°58′13.3″ E: 008°52′34.9″	Hot	24.0 °C	1–6 mm	Olive trees	Agriculture
5	N: 36°58′12.8″ E: 008°52′34.7″	Hot	24.0 °C	1–6 mm	Pine trees	Forest

**Table 2:** Physical and chemical characteristics of soil samples.

Site	Type	Texture	pH	Organic Matter (%)	Total Carbon (%)	Total Nitrogen (mg/L)	Total Phosphorous (ppm)
1	Forest	Sandy clay	7.05	13.21	7.65	0.52	67.19
2	Forest	Sandy loam	6.50	7.02	4.06	0.39	72.51
3	Forest	Sandy loam	6.56	11.27	6.49	0.47	68.86
4	Agriculture	Sandy loam	6.90	4.38	2.53	0.30	66.12
5	Forest	Sandy	6.60	1.07	0.62	0.28	66.69

**Table 3:** Phosphate solubilization index of PSB isolates (mean ± confidence interval, n = 3). Same letters indicate that the difference between the means is not statistically significant.

PSB	SI in NBRIP	SI in PVK
*Pseudomonas putida*	3.53 ± 0.05 a	2.14 ± 0.03 e
*Pseudomonas fluorescens*	3.82 ± 0.05 c	3.51 ± 0.05 a
*Pseudomonas taiwanensis*	3.64 ± 0.05 a	3.12 ± 0.03 f
*Stenotrophomonas maltophilia*	2.83 ± 0.02 d	2.31 ± 0.02 b
*Pantoea agglomerans*	2.35 ± 0.02 b	2.98 ± 0.03 g

**Table 4:** Correlation of the average concentration of phosphate solubilized by the isolates with soil properties (Pearson’s correlation).

Soil Property	Correlation Coefficient	*p*-Value
pH	−0.759	0.137
Organic Matter (%)	0.210	0.734
Total Carbon (%)	0.208	0.737
Total Nitrogen (mg/L)	0.236	0.703
Total Phosphorous (ppm)	0.954	0.012

## Data Availability

The data used to support the finding of this study are available upon request.
